# Occupational patterns and risks of occupational accidents with biological material: A cross-sectional study, Brazil, 2015-2019

**DOI:** 10.1590/S2237-96222025v34e20250051.en

**Published:** 2025-10-27

**Authors:** Júlia de Albuquerque Índio do Brasil, Letícia Martins Raposo

**Affiliations:** 1 Universidade Federal do Estado do Rio de Janeiro, Centro de Ciências Exatas e Tecnologia, Rio de Janeiro, RJ, Brazil

**Keywords:** Accidents, Occupational, Health Personnel, Containment of Biohazards, Biological Contamination, Cluster Analysis, Accidentes de Trabajo, Personal de Salud, Contención de Riesgos Biológicos, Contaminación Biológica, Análisis por Conglomerados

## Abstract

**Objectives:**

To characterize the profile of occupational accidents involving biological material in Brazil and identify patterns associated with these events.

**Methods:**

This study employed a cross-sectional design using secondary data from occupational accidents registered in the Notifiable Health Conditions Information System (SINAN) between 2015 and 2019. Absolute and relative frequencies were used to describe demographic variables, working conditions, and types of exposure. Multiple correspondence and hierarchical clustering analyses were employed, using values of the v-test to evaluate associations between categories and groupings.

**Results:**

Between 2015 and 2019, Brazil reported 69,663 cases of occupational accidents involving exposure to biological materials. Nursing professionals accounted for 70.9% of the reported cases, with a predominance of women (82.0%) and workers in the 30-39 years age group (36.7%). Most accidents occurred in the Southeast (46.0%) and involved percutaneous exposure (88.3%), with needles being the main causative agent (92.1%). Significant differences were identified between the three occupational groups. Group 1 (cleaning workers) had a low education level, male predominance, high percutaneous exposure, and low vaccination rates against hepatitis B. Group 2 (nursing) stood out for its high vaccination rates, female predominance, and accidents related to medication administration. Group 3 (dentistry/medicine) had higher education, a predominance of young people, and accidents involving glass and blades, as well as mucosal exposure, with a high usage rate of personal protective equipment.

**Conclusion:**

Three patterns of occupational accidents with exposure to biological material were identified in Brazil, differentiated by occupation, education level, vaccination profile, and type of exposure.

## Resumen

**Objetivos:**

Caracterizar el perfil de los accidentes de trabajo con material biológico en Brasil e identificar patrones asociados a estos eventos.

**Métodos:**

Se trató de un estudio transversal con datos secundarios de accidentes laborales registrados en el Sistema de Información de Agravios de Notificación entre 2015 y 2019. Las variables demográficas, las condiciones laborales y los tipos de exposición se describieron mediante frecuencias absolutas y relativas. Se emplearon análisis de correspondencia múltiple y análisis de agrupamiento jerárquico, utilizando valores de v-test para evaluar asociaciones entre categorías y agrupamientos.

**Resultados:**

Entre 2015 y 2019, Brasil registró 69.663 casos de accidentes laborales con exposición a material biológico. Los profesionales de enfermería representaron el 70,9% de los casos notificados, con predominio de mujeres (82,0%) y trabajadores de 30 a 39 años (36,7%). La mayoría de los accidentes ocurrió en la región Sudeste (46,0%) e involucró exposición percutánea (88,3%), siendo las agujas el principal agente causante (92,1%). Se identificaron diferencias significativas entre tres grupos ocupacionales. El grupo 1 (trabajadores de limpieza) presentó baja escolaridad, predominio masculino, alta exposición percutánea y baja vacunación contra la hepatitis B. El grupo 2 (enfermería) se destacó por alta cobertura vacunal, mayoría femenina y accidentes relacionados con la administración de medicamentos. El grupo 3 (odontología/medicina) mostró mayor nivel educativo, predominio de jóvenes y accidentes con vidrio y láminas, así como exposición mucosa, con alta adherencia al uso de equipos de protección individual.

**Conclusión:**

Se identificaron tres patrones de accidentes de trabajo con exposición a material biológico en Brasil, diferenciados por ocupación, nivel educativo, perfil de vacunación y tipo de exposición.

**Ethical aspects**

This research used public domain anonymized databases.

## Introduction 

Occupational accidents are events that occur during work activities, resulting in physical or functional harm to workers and negatively impacting both individual well-being and the economic efficiency of organizations, due to reduced workforce and decreased productivity (1). Among the various types of accidents, those related to exposure to biological material are a significant concern for global public health, due to the risk of transmitting infectious diseases through contaminated body fluids or by percutaneous or mucosal exposure (2,3).

The main pathogens associated with occupational accidents involving biological material include hepatitis B and C viruses and Human Immunodeficiency Virus (HIV) (4). Although all pose significant risks to the physical health of workers, HIV stands out for its significant psychosocial impacts, due to the stigma and discrimination associated with positive serostatus (5,6). Although health professionals are admittedly the most vulnerable to such exposures, workers in other sectors, such as cleaning and conservation, are also exposed to biological materials, highlighting the breadth and complexity of this problem (1,2). 

About 2.78 million individuals die annually as a result of work-related accidents, which indicates significant economic, social, and family impact associated with these events (2,7). In Brazil, the Ministry of Health has established specific protocols to care for workers exposed to biological materials, implementing treatment flows and clear guidelines for the notification and management of these cases within health services. Ordinance No. 777/2004 established the inclusion of 11 work-related diseases in the Notifiable Health Conditions Information System (SINAN), covering accidents with biological material (8).

Considering this regulatory and institutional context, this analysis aimed to provide insights that contribute to the development and implementation of more effective prevention strategies, promoting work environments that ensure better safety and health conditions for workers. This study aimed to characterize the epidemiological profile of occupational accidents related to exposure to biological material in Brazil and identify patterns associated with these events. 

## Methods 

### Study design

This was a cross-sectional, quantitative study conducted through the analysis of notifications of occupational accidents involving exposure to biological material in Brazil between January 2015 and December 2019.

### Setting 

The study was conducted using data collected from all regions of Brazil in August 2023. The information analyzed was obtained through the SINAN, considering all notifications registered in the system between January 2015 and December 2019.

This approach enabled the analysis of the distribution of occupational accidents involving biological material, reflecting the diverse work scenarios present in a country with an estimated population of 212.6 million inhabitants, distributed across 5,571 municipalities, 26 states, and the Federal District, as of August 2024.

### Participants 

The study included notifications of workers exposed to biological materials during the exercise of their occupations, which occurred in Brazil between 2015 and 2019.

### Variables 

The variables analyzed in this study were: gender (Male, Female); age group (in years: 16-29, 30-39, 40-49, 50-59, 60+); race/skin color (White, Brown, Black, Asian, Indigenous); education level (up to high school, incomplete higher education, complete higher education); region of residence (North, Northeast, Midwest, Southeast, South); occupation (health laboratory assistants; dental surgeons/dental technicians; nurses/nursing technicians and assistants; pharmacists; doctors; domestic service workers, waste collection, cleaning and conservation of public areas, dye and laundry workers); work relationship (formal, informal); working time in current occupation (>6 months, 6-11 months, 1-2 years, 3-5 years, 6+); type of exposure (percutaneous, intact skin, non-intact skin, mucous membrane); circumstance of the accident (medication administration, venous/arterial puncture, disposal and handling of materials, surgical/dental/laboratory procedures); causative agent of the accident (needles/catheter intracath, glass/blade/lancet); use of personal protective equipment (gloves, apron, goggles, mask, face shield, boots); three doses of hepatitis B vaccination (yes, no); and patient identification-known source (yes, no).

### Data source and measurement

The data used in this study were extracted from the SINAN, available on the portal of the Brazilian National Health System Information Technology Department (DATASUS) (https://datasus.saude.gov.br/). Accidents registered among workers in the formal and informal labor market, with a minimum age of 16 years, were analyzed.

The variables of interest were extracted directly from the notifications registered in the SINAN. The occupations of the workers involved in the accidents were classified according to the Brazilian Classification of Occupations (CBO), allowing for the systematic categorization of the ten occupations most frequently associated with accidents involving biological materials.

### Bias control

Records with inconsistencies, including inaccurate, incomplete information, or with fields filled in as “ignored,” were identified and excluded from the final analysis.

### Bias 

This study, based on secondary data from the SINAN, was subject to: (I) information bias, due to incorrect or “ignored” records, mitigated by the exclusion of notifications with incomplete data; and (ii) selection bias, since only officially notified accidents were considered, which may underestimate events in informal sectors or with low usage rate of the system.

### Study size

No previous sample estimation was performed. All notifications from 2015 to 2019 were included and then excluded according to the specified criteria.

### Statistical methods

The data were initially organized in Microsoft Excel (Microsoft Corporation, Microsoft Excel 365, Redmond, United States) and then exported to the R software (R Foundation for Statistical Computing, Vienna, Austria), where statistical analyses were performed. Both absolute and relative frequencies are used to describe categorical variables. To identify patterns in occupational accidents involving biological material, two complementary techniques were applied: multiple correspondence analysis, used to treat categorical variables, and hierarchical grouping analysis, based on the main components extracted from multiple correspondence analysis.

Due to the large volume of data and the computational demands associated with the techniques used, a sample of 3 thousand observations was drawn for each year of the analyzed period (2015-2019), resulting in 15 thousand observations for the final analysis. This strategy enabled the maintenance of the sample’s representation and preserved the robustness of the results.

Cluster analysis was implemented using the HCPC function of the FactoMineR package (9), available at R. The determination of the optimal number of clusters was based on the inertia criterion. This criterion considered the reduction in total inertia when forming the clusters, balancing intragroup homogeneity and simplicity of the solution, resulting in an optimized and clear division of the data.

To identify the variables that contributed most to the formation of the groups, the following was applied: a v-test (9), with a significance level of 5%, as determined through the analysis performed with the HCPC function. The v-test evaluated the statistical association between variables or categories and the groups formed, measuring the deviation of the observed mean from the expected mean, under the null hypothesis that the variables or categories were randomly distributed among the groups. 

This statistic allowed for the identification of the most characteristic variables or categories within each group, highlighting those that were significantly overrepresented or underrepresented. High or low values (in absolute terms) of the v-test indicated a relevant discrepancy between the observed and expected means or proportions, signaling significant contributions to the definition of a grouping. Categories with v-test ≤5 were classified as low representation; between 5 and 10, as medium representation; and above 10, as high representation. 

The groupings were analyzed using two complementary approaches. The first quantified the proportion of each category in each cluster, while the second evaluated the proportion of observations from each cluster in each category.

### Data availability and cleaning methods

The data used in this study are publicly available and were provided by the Ministry of Health. Before analysis, these data underwent a cleaning process, which involved removing duplicates and correcting inconsistencies, followed by quality checks to ensure the integrity and consistency of the variables of interest. Data processing and analysis were conducted using the R software. The database used in the research is available at: https://zenodo.org/doi/10.5281/zenodo.15310979 (10).

## Results 

Between 2015 and 2019, Brazil reported 69,663 cases of occupational accidents involving exposure to biological materials, a rise from 12,059 in 2015 to 15,480 in 2019.

The majority of injured workers belonged to the 30-39 year age group (36.7%), followed by the 16-29 year age group (30.7%), with a female predominance (82.0%) and a higher prevalence among White individuals (61.3%). In terms of education level, 64.9% completed high school, while 27.4% completed higher education (Table 1).

**Table 1:** Distribution of demographic and occupational characteristics (absolute and relative frequencies) of cases of occupational accidents with exposure to biological material. Brazil, 2015-2019 (n=69,663)

Characteristics	n=69.663 (%)
**Year of notification**	
2015	12,059 (17.3)
2016	12,739 (18.3)
2017	14,276 (20.5)
2018	15,109 (21.7)
2019	15,480 (22.2)
**Age group** (years)	
16-29	21,386 (30.7)
30-39	25.539 (36,6)
40-49	14,814 (21.3)
50-59	6,598 (9.5)
1	1,326 (1.8)
Sex	
Female	57,142 (82.0)
Male	12,521 (18.0)
**Race/skin color**	
White	42,717 (61.3)
Brown	21,402 (30.7)
Black	4,872 (7.0)
Asian	541 (0.8)
Indigenous	131 (0.2)
**Education level**	
Up to high school	45,223 (64.9)
Incomplete higher education	5,328 (7.6)
Higher education	19,112 (27.5)
**Region of Brazil**	
North	3,152 (4.5)
Northeast	9,075 (13.0)
Central-West	6,734 (9.7)
Southeast	32,013 (46.0)
South	18,689 (26.8)
Occupation	
Health laboratory assistants	1,723 (2.5)
Dental surgeons/dental technicians	4,691 (6.7)
Nurses/nursing technicians, and assistants	49,375 (70.9)
Pharmacists	825 (1.2)
Doctors	4,636 (6.6)
Domestic service workers/waste collection, cleaning and maintenance of public areas/dye and laundry workers	8,413 (12.1)
**Employment relationship**	
Formal	61,702 (88.6)
Informal	7,961 (11.4)
**Time in occupation**	
>6 months	11,880 (17.1)
6-11 months	7,017 (10.1)
1-2 years	15,740 (22.6)
3-5 years	14,164 (20.3)
6+	20,862 (29.9)
**Type of exposure**	
Percutaneous	61,529 (88.3)
Intact skin	21,392 (30.7)
Non-intact skin	2,758 (4.0)
Mucous membrane	1,325 (1.9)
**Circumstances of the accident**	
Medication administration	22,273 (32.0)
Venous/arterial puncture	12,176 (17.5)
Disposal and handling of materials	23,436 (33.6)
Surgical/dental/laboratory procedure	11,778 (16.9)
**Causative agent**	
Needle/intracath	64,159 (92.1)
Glass/blade/lancet	5,504 (7.9)
**Use of personal protective equipment**	
Gloves	55,554 (79.7)
Apron	32,489 (46.6)
Glasses	15,271 (21.9)
Mask	20,129 (28.9)
Face shield	3,900 (5.6)
Boots	13,322 (19.1)
Received all three doses of the hepatitis B vaccine	66,266 (95.1)
Known source patient	50,712 (72.8)

The Southeast concentrated 46.0% of cases, followed by the South (26.8%) and the Northeast (13.0%). Nursing professionals, technicians, and assistants represented 70.9% of the notifications. Most workers had a formal employment relationship (88.6%), and 29.9% had been in the occupation for six years or more. Percutaneous exposure was predominant (88.3%), with needles and intracaths being the main causative agents (92.1%) (Table 1).

Regarding the use of personal protective equipment, 79.7% wore gloves, and 46.6% wore an apron. Vaccination coverage against hepatitis B was high, with 95.1% of individuals receiving all three doses. Additionally, 72.8% of cases involved patients with a known source (Table 1).

**Figure 1:**
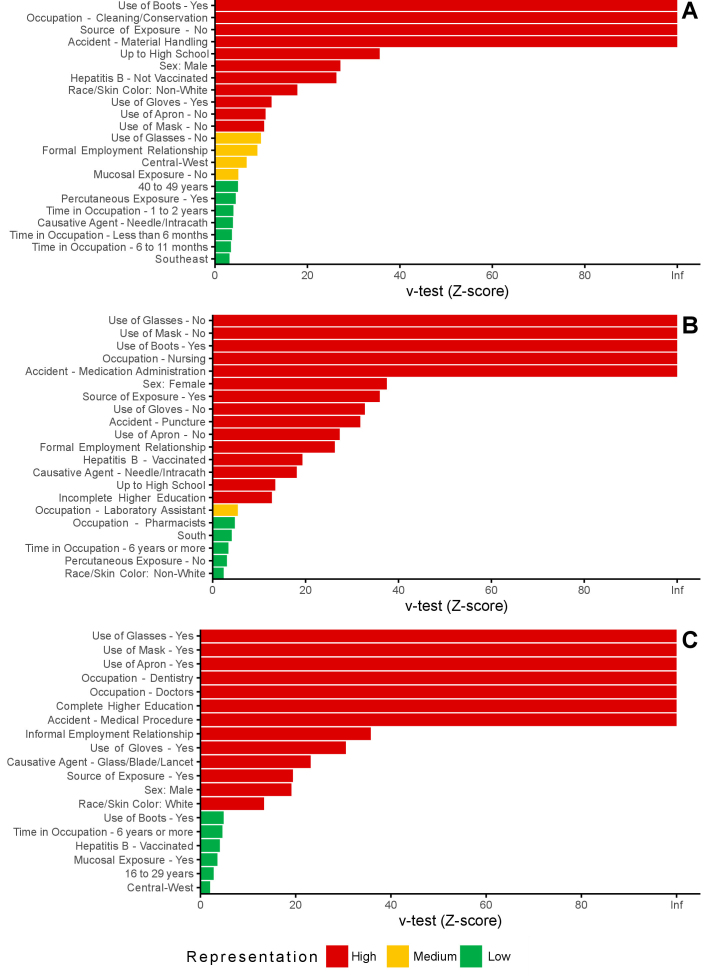
Representation of the variable categories in groups of occupational accident cases with exposure to biological material, according to values of v-test: ≤5 (low representation), between 5 and 10 (average representation), and >10 (high representation). Brazil, 2015-2019

The analysis of the groups revealed distinctions between the three identified groups, both in terms of demographic and occupational characteristics, as well as in relation to the circumstances of accidents involving exposure to biological material (Table 2; Figure 1).

**Table 2:** Characterization of cases of occupational accidents with exposure to biological material in three groups (Group 1 – cleaning and conservation; Group 2 – nursing; Group 3 – medicine and dentistry), considering only categories with statistically significant association (p-valor<0.05). Brazil, 2015-2019 (n=15,000)

		Group 1		Group 2		Group 3	
Categories	Overall^a^	Category/ group^b^	Group/ category^c^	Category/ group^b^	Group/ category^c^	Category/ group^b^	Group/ category^c^
**Age group** (years)							
16-29	30.5	12.8	28.4			20.2	32.7
30-39	36.6	12.6	33.6				
40-49	21.6	16.5	25.8			15.3	17.5
Sex							
Female	82.1	9.8	58.7	74.3	90.5	15.8	69.0
Male	17.9	31.6	41.3	35.7	9.5	32.6	31.0
**Race/skin color**							
White	62.4	9.8	44.4	68.1	63.0	22.1	73.1
Non-White	37.6	20.3	55.6	66.2	37.0	13.5	26.9
**Education level**							
Up to high school	64.7	20.3	95.4	71.2	68.4	8.5	29.2
Incomplete higher education	7.4	4.0	2.1	83.7	9.2	12.3	4.8
Higher education	27.9	1.2	2.5	54.2	22.4	44.6	65.9
Region							
Central-West	10.0	19.8	14.4	59.4	8.8	20.8	11.0
Southeast	45.3	14.7	48.5			17.6	42.4
South	27.2	10.4	20.5	70.0	28.2		
Occupation							
Health laboratory assistants	2.4	6.0	1.1	80.0	2.9	14.0	1.8
Dental surgeons/dental technicians	6.6	0.9	0.4	8.7	0.9	90.4	31.7
Nurses/nursing technicians and assistants	71.2	2.5	13.2	88.8	93.9	8.6	32.5
Pharmacists	1.3	5.6	0.5	82.6	1.6	11.8	0.8
Doctors	6.7	0.2	0.1	6.8	0.7	93.0	33.0
Domestic service workers/waste collection, cleaning and maintenance of public areas/dye and laundry workers	11.8	99.0	84.7	0.8	0.1	0.2	0.1
**Type of employment relationship**							
Formal	88.7	14.6	94.2	71.1	93.6	14.3	67.2
Informal	11.3	7.0	5.8	38.2	6.4	54.8	32.8
**Time in occupation**							
>6 months	17.4	16.0	20.3				
6-11 months	10.1	16.7	12.3			14.7	7.9
1-2 years	22.0	15.9	25.4	65.5	21.4		
6+	30.1	9.5	20.8	69.4	30.9	21.1	33.7
**Type of exposure**							
Percutaneous	88.4	14.2	91.2	67.0	87.8		
Mucous membrane	1.7	4.3	0.5			27.9	2.5
**Circumstances of the accident**							
Medication administration	32.1	0.9	2.1	94.5	45.0	4.6	7.9
Venous/arterial puncture	17.3	0.5	0.6	91.6	23.5	7.9	7.3
Disposal and handling of materials	33.5	39.9	97.1	53.1	26.4	7.0	12.4
Surgical/dental/laboratory procedure	17.1	0.2	0.2	19.9	5.1	79.9	72.4
**Causative agent**							
Needle/intracath	92.1	14.1	94.2	69.5	95.0	16.4	80.3
Glass/blade/lancet	7.9	10.1	5.8	42.9	5.0	47.0	19.7
**Use of personal protective equipment**							
Gloves	79.7	15.4	89.1	61.6	72.8	23.1	97.5
Apron	46.9	10.5	35.8	56.3	39.2	33.2	82.6
Glasses	21.8	8.7	13.7	34.5	11.2	56.8	65.6
Mask	28.7	9.2	19.2	37.6	16.0	53.2	81.1
Boots	19.2	45.5	63.5	32.4	9.2	22.1	22.5
**Three doses of the hepatitis B vaccine**							
Not vaccinated	4.9	53.0	19.1	33.6	2.5	13.4	3.5
Vaccinated	95.1	11.7	80.9	69.2	97.5	19.1	96.5
**Known exposure source**	72.8	1.6	8.4	76.0	82.1	22.4	86.7

^a^Overall proportion (%) before grouping; ^b^Proportion (%) of category belonging to the group; ^c^Proportion (%) of the category within the group.

Values of v-test by category in each group (a: Group 1-cleaning and conservation; B: Group 2-nursing; and C: Group 3-medicine and dentistry); “Inf” indicates infinite v-test values resulting from a zero denominator in standardized calculation.

Group 1 concentrated mostly male workers (41.3% compared to 17.9% of the overall proportion) who were between 40 and 49 years of age (25.8%, although they represented 21.6% of the total sample), non-White individuals (55.6%) and with education level up to high school (95.4%). This group was predominantly composed of individuals involved in cleaning and conservation activities (84.7%), with the majority having a formal employment relationship (94.2%). Accidents in this group were related to material handling (97.1%) and percutaneous exposure (91.2%). The use of personal protective equipment, such as aprons, masks, and glasses, was low, as was vaccination coverage against hepatitis B – 19.1% of participants had incomplete vaccination schedules (this percentage was 4.9% in the overall proportion), accounting for 53.0% of total cases in this condition. A high proportion of workers were unaware of the source of their exposure.

Group 2 consisted predominantly of women (90.5%), nursing professionals (93.9%), with education level up to high school (68.4%), and White individuals (63.0%). Most of these individuals worked under a formal employment regime (93.5%) and had prolonged time in the occupation (30.9% with more than six years of experience). This group was associated with accidents during medication administration (45.0%) and exhibited a high rate of vaccination coverage against hepatitis B (97.5% with three doses), as well as a high degree of knowledge regarding the source of exposure (82.1%). However, a low usage rate of personal protective equipment was observed, with lower proportions in all items evaluated when compared to those in the overall sample.

Group 3 was characterized by a higher proportion of young individuals, aged between 16 and 29 years (32.7%), and by the relative predominance of male workers compared to the overall sample (31.0% versus 17.9%). The high percentage of participants with complete higher education (65.9%) stood out. This group was predominantly composed of dental and medical professionals, with each category accounting for more than 30.0% of the group members, in contrast to less than 7.0% of each category in the overall sample. Significant performance was observed under informal employment relationships (32.8%). Accidents related to the use of sharp materials, such as glass and blades, predominated (19.7%, compared to 7.9% in the overall sample), as well as exposure to mucous membranes (2.5% compared to 1.7% in the total sample). There was a high usage of personal protective equipment, exceeding the general average frequency. Most of the injured workers in this group reported knowing the source of exposure (86.7%).

## Discussion 

The findings of this study suggest that a complex interaction exists between sociodemographic and labor-related factors, influencing the risk of occupational accidents involving biological materials in Brazil. The existence of marked inequalities in access to protection and the adoption of preventive practices, which vary significantly by occupational group, was observed. The analysis enabled the identification of three distinct groups of workers affected by such accidents.

This study presents limitations due to the use of secondary data, which is subject to underreporting and inconsistencies in surveillance systems, potentially compromising the accuracy of the findings. The absence of important variables, such as biosafety training and exposure time, restricted the analysis of factors associated with accidents. The heterogeneity of occupational contexts and regional policies may also have influenced the results, limiting their generalization. Additionally, the analysis was limited to cases reported during the study period, without considering temporal variations. Despite these limitations, the findings contribute to a comprehensive understanding of occupational risks associated with exposure to biological materials and reinforce the need for continuous and effective preventive measures.

Group 1 was composed mostly of non-White men, with education level up to high school, working in cleaning and conservation services -a profile similar to that observed in a hospital in the inland region of São Paulo, where workers in the sanitation sector had low education level, reduced income and predominance of self-declared Black and Brown individuals (11). This configuration reflects structural inequalities in the labor market, in which Black individuals, especially those with a lower education level, occupy more precarious positions and are exposed to risks (12). The low usage rate of personal protective equipment, incomplete vaccination coverage, and lack of knowledge about the source of exposure observed in this group indicate a greater vulnerability to occupational risks associated with biological materials.

Most accidents were associated with the improper handling of sharp materials, often improperly disposed of by health professionals (13-15). In six hospitals in São Luís, it was observed that 13.57% of the cleaning workers had suffered occupational accidents, mostly with sharp materials. Individuals with incomplete high school education presented a significantly higher risk, which evidenced the impact of schooling on compliance with biosafety practices (16).

Mitigating these risks requires the availability of appropriate safety devices and the continuous provision of training on the correct use and disposal of sharp materials, in addition to the effective implementation of appropriate segregation of health services waste (17). Failure to use personal protective equipment was associated with a five times higher probability of accidents among urban cleaning workers in São Luís (16). In Goiânia, hospital sanitation workers reported high rates of glove and rubber boot use, while the use of aprons, masks, and eye protection was substantially lower, despite the recognition of their importance for safety (18). A similar pattern was identified in Group 1 of this analysis.

The proper use of personal protective equipment is a fundamental measure in preventing accidents involving biological material, serving as a physical barrier against cross-contamination and substantially reducing the risk of occupational infections (19). Vaccination against hepatitis B represents another essential protective strategy for exposed professionals, as it is effective in reducing the risk of infection by the virus (20,21). In Goiânia, approximately one third of hospital sanitation workers had not completed the vaccination schedule, although the importance of immunization is recognized (18) – a pattern also observed among the members of Group 1 in this investigation.

Group 2 was composed mostly of White women, with education level up to high school, working in the nursing field, under a formal employment relationship, and with prolonged experience in the occupation. This group demonstrated high vaccination coverage against hepatitis B and a high degree of knowledge about the source of exposure. Despite these protective factors, a low usage rate of personal protective equipment was observed, with levels lower than those verified in the overall sample for all items evaluated. 

This group presented a high frequency of percutaneous needle injuries, which occurred mostly during medication administration procedures (22) - an activity intrinsically associated with the risk of contact with sharp materials and biological fluids. In Minas Gerais, between October 2014 and May 2016, intramuscular medication administration was identified as the activity involved in 16.4% of reported percutaneous exposures (23). Similarly, in Pernambuco, it was observed that 62.7% of accidents with biological material were related to medication administration (24).

Group 2 accounted for the majority of registered cases, a pattern also observed at the national level. Between 2018 and 2022, 54.4% of the 329,176 occupational accidents with exposure to biological material occurred among nursing professionals (25). This finding was consistent with the majority female composition of the nursing workforce in Brazil. Women represent a large majority in this category, contributing to the high level of female participation among injured workers (26). This profile is confirmed in regional studies: in Minas Gerais, 91.8% of the exposed professionals were female nursing professionals (23); in Pernambuco, between 2009 and 2019, 83.6% of cases also involved female professionals (24).

The use of gloves was reported among nursing professionals, but its frequency was lower than that observed in the overall sample. The use of other protective devices, such as glasses and masks, was even more restricted, which highlighted gaps in the biosafety practices of this group (2,3,19). 

In most cases, the source of exposure was identified, allowing adequate serological follow-up. This finding is confirmed by data from Minas Gerais, where the serological status of the source patient was recorded in most reports of accidents among nursing professionals (23). In addition, Group 2 had considerable vaccination coverage against hepatitis B. In Pernambuco, 62.7% of the injured workers had the complete vaccination schedule against this disease (24).

Group 3 included, in a proportionally higher way compared to the overall sample, professionals from dentistry and medicine. In this group, the inadequate handling of sharp materials, such as glass fragments (possibly from ampoules), blades, and scalpels, represents a significant vulnerability in terms of biosafety. There was a high prevalence of accidents involving sharp materials among health care workers, more frequently among dental professionals, due to the performance of invasive procedures and the constant manipulation of sharp instruments (27). In two dental education institutions in Brazil, 25.3% of students suffered accidents of this type during training, with a higher incidence in the final periods of the course, when the clinical exposure is more intense (28). 

Although Group 3 had a higher proportion of men compared to the overall sample, female representation remained significant. Such information does not imply a causal association between gender and neglect, but reflects the specific occupational composition of the analyzed group. In addition, this group stood out for the higher concentration of informal employment relationships, including scholarships, internships, residency programs, and self-employment, types of contracts often observed between doctors and dentists in the Brazilian context (29,30).

Despite the importance of the topic, the data analyzed show a critical gap regarding adherence to post-exposure prophylaxis (PEP) for HIV, which is essential in the immediate management of accidents with biological risk. The absence of systematized information on the indication, initiation, continuity, and conclusion of the conduct adopted by health services hinders the evaluation of the conduct, compromising institutional monitoring and the planning of strategies that guarantee equitable access and adequate follow-up. Qualitative studies with primary data are necessary to deepen the understanding of the factors that influence compliance with post-exposure prophylaxis (PEP), including the level of knowledge of professionals, the structure of services, and institutional barriers.

The results of this study suggest that occupational accidents involving biological materials in the Brazilian context are the outcome of a complex interplay between sociodemographic, occupational, and institutional factors. In this scenario, the greater vulnerability of specific groups, such as cleaning workers and nursing professionals, whose work practices expose them more frequently to risk situations, especially in the handling of sharp materials and insufficient compliance with biosafety standards. This framework reinforced the need for structured interventions that articulate actions of permanent health education, equitable access to personal protective equipment, strengthening policies of surveillance in occupational health, and monitoring compliance with regulatory standards. 
