# Supplementary material for: Occupational patterns and risks of occupational accidents with biological material: A cross-sectional study, Brazil, 2015-2019
Source: Epidemiol Serv Saude. 2025 Oct 27;34:e20250051. doi: 10.1590/S2237-96222025v34e20250051.en (PMC12560222; doi:10.1590/S2237-96222025v34e20250051.en)
Supplement: Translation

# Translation: Padrões ocupacionais e riscos em acidentes de trabalho com material biológico: estudo transversal, Brasil, 2015-2019

**DOI:** 10.1590/S2237-96222025v34e20250051.pt

**Authors:** Júlia de Albuquerque Índio do Brasil, Letícia Martins Raposo

**Affiliations:** 1 Universidade Federal do Estado do Rio de Janeiro, Centro de Ciências Exatas e Tecnologia, Rio de Janeiro, RJ, Brasil

**Keywords:** Acidentes de Trabalho, Pessoal de Saúde, Contenção de Riscos Biológicos, Contaminação Biológica, Análise por Conglomerados

## Abstract

**Objetivos:**

Caracterizar o perfil dos acidentes de trabalho envolvendo material biológico no Brasil e identificar padrões associados a esses eventos.

**Métodos:**

Tratou-se de estudo transversal com dados secundários de acidentes de trabalho registrados no Sistema de Informação de Agravos de Notificação entre 2015 e 2019. As variáveis demográficas, as condições laborais e os tipos de exposição foram descritos por frequências absolutas e relativas. Foram empregadas a análise de correspondência múltipla e análise de agrupamento hierárquico, utilizando valores de v-test para avaliar associações entre as categorias e os agrupamentos.

**Resultados:**

Entre 2015 e 2019, o Brasil registrou 69.663 casos de acidentes de trabalho com exposição a material biológico. Profissionais de enfermagem representaram 70,9% dos casos notificados, com predominância de mulheres (82,0%) e trabalhadores na faixa etária 30-39 anos (36,7%). A maioria dos acidentes ocorreu no Sudeste (46,0%) e envolveu exposição percutânea (88,3%), com agulhas sendo o principal agente causador (92,1%). Diferenças significativas foram identificadas entre três grupos ocupacionais. O grupo 1 (trabalhadores de limpeza) apresentou baixa escolaridade, predominância masculina, alta exposição percutânea e baixa vacinação contra hepatite B. O grupo 2 (enfermagem) destacou-se por alta vacinação, maioria feminina e acidentes ligados à administração de medicamentos. O grupo 3 (odontologia/medicina) teve maior escolaridade, predominância de jovens e acidentes com vidro e lâmina e exposição mucosa, com alta adesão a equipamentos de proteção individual.

**Conclusão:**

Foram identificados três padrões de acidentes de trabalho com exposição a material biológico no Brasil, diferenciados por ocupação, nível de escolaridade, perfil vacinal e tipo de exposição.

**Aspectos éticos**

Esta pesquisa utilizou bancos de dados de domínio público e anonimizados.

## Introdução 

Acidentes ocupacionais são eventos ocorridos no desempenho de atividades laborais que resultam em danos físicos ou funcionais aos trabalhadores, impactando negativamente tanto o bem-estar individual quanto a eficiência econômica das organizações, em virtude da redução da força de trabalho e da diminuição da produtividade (1). Entre os diversos tipos de acidentes, aqueles relacionados à exposição a material biológico destacam-se como uma preocupação significativa para a saúde pública global, devido ao risco de transmissão de doenças infectocontagiosas por meio de fluidos corporais contaminados ou por exposição percutânea ou de mucosa (2,3).

Os principais agentes patogênicos associados a acidentes de trabalho envolvendo material biológico incluem os vírus das hepatites B e C e o da imunodeficiência humana (HIV) (4). Embora todos representem riscos significativos à saúde física dos trabalhadores, o HIV destaca-se por seus impactos psicossociais acentuados, devido ao estigma e à discriminação associados à soropositividade (5,6). Apesar de os profissionais de saúde serem reconhecidamente os mais vulneráveis a tais exposições, trabalhadores de outros setores, como os de limpeza e conservação, também estão sujeitos à exposição a materiais biológicos, o que evidencia a amplitude e complexidade do problema (1,2). 

Cerca de 2,78 milhões de indivíduos falecem anualmente em decorrência de acidentes relacionados ao trabalho, o que indica impacto econômico, social e familiar significativo associado a esses eventos (2,7). No Brasil, o Ministério da Saúde estabeleceu protocolos específicos para atender trabalhadores expostos a materiais biológicos, com a implementação de fluxos de tratamento e diretrizes claras para a notificação e o manejo desses casos nos serviços de saúde. A Portaria nº 777/2004 estabeleceu a inclusão de 11 agravos relacionados ao trabalho no Sistema de Informação de Agravos de Notificação, abrangendo os acidentes com material biológico (8).

Considerando esse cenário normativo e institucional, esta análise buscou oferecer subsídios que contribuam para o desenvolvimento e a implementação de estratégias preventivas mais eficazes, promovendo ambientes de trabalho que assegurem melhores condições de segurança e saúde aos trabalhadores. Este estudo teve como objetivos caracterizar o perfil epidemiológico dos acidentes de trabalho relacionados à exposição a material biológico no Brasil e identificar padrões associados a esses eventos. 

## Métodos 

### Delineamento do estudo

Tratou-se de estudo transversal e quantitativo, conduzido por meio da análise das notificações de acidentes de trabalho com exposição a material biológico no Brasil entre janeiro de 2015 e dezembro de 2019.

### Contexto 

O estudo foi conduzido com base em dados abrangendo todas as regiões do Brasil, coletados em agosto de 2023. As informações analisadas foram obtidas por meio do Sistema de Informação de Agravos de Notificação, considerando todas as notificações documentadas no sistema entre janeiro de 2015 e dezembro de 2019.

Essa abordagem permitiu analisar a distribuição dos acidentes de trabalho que envolveram material biológico e refletiram os cenários laborais diversificados presentes em um país com população estimada de 212,6 milhões de habitantes, distribuídos em 5.571 municípios, 26 estados e o Distrito Federal, em agosto de 2024.

### Participantes 

O estudo incluiu notificações de trabalhadores e trabalhadoras expostos a materiais biológicos durante o exercício de suas ocupações, ocorridas no Brasil, entre 2015 e 2019.

### Variáveis 

As variáveis analisadas neste estudo foram: sexo (masculino, feminino); faixa etária (em anos: 16-29, 30-39, 40-49, 50-59, 60+); raça/cor da pele (branca, parda, preta, amarela, indígena); escolaridade (até ensino médio, ensino superior incompleto, ensino superior completo); região de residência (Norte, Nordeste, Centro-Oeste, Sudeste, Sul); ocupação (auxiliares de laboratório da saúde; cirurgiões-dentistas/técnicos de odontologia; enfermeiros/técnicos e auxiliares de enfermagem; farmacêuticos; médicos; trabalhadores dos serviços domésticos, coleta de resíduos, limpeza e conservação de áreas públicas, tintureiros e lavadeiros); vínculo de trabalho (formal, informal); tempo de trabalho na ocupação atual (>6 meses, 6-11 meses, 1-2 anos, 3-5 anos, 6+); tipo de exposição (percutânea, pele íntegra, pele não íntegra, mucosa); circunstância do acidente (administração de medicação, punção venosa/arterial, descarte e manuseio de materiais, procedimentos cirúrgicos/odontológicos/laboratoriais); agente causador do acidente (agulhas/cateter intracath, vidro/lâmina/lanceta); uso de equipamento de proteção individual (luvas, avental, óculos, máscara, proteção facial, botas); três doses de vacinação contra hepatite B (sim, não); e identificação de paciente-fonte conhecida (sim, não).

### Fontes de dados e mensuração

Os dados utilizados neste estudo foram extraídos do Sistema de Informação de Agravos de Notificação, disponível no portal do Departamento de Informática do Sistema Único de Saúde (https://datasus.saude.gov.br/). Foram analisados acidentes registrados entre trabalhadores do mercado formal e informal, com idade mínima de 16 anos.

As variáveis de interesse foram extraídas diretamente das notificações registradas no Sistema de Informação de Agravos de Notificação. As ocupações dos trabalhadores envolvidos nos acidentes foram classificadas de acordo com a Classificação Brasileira de Ocupações, permitindo a categorização sistemática das dez ocupações mais frequentemente relacionadas a acidentes com material biológico.

### Controle de viés

Registros com inconsistências, incluindo informações imprecisas, incompletas ou com campos preenchidos como “ignorado”, foram identificados e excluídos da análise final.

### Viés 

Este estudo, baseado em dados secundários do Sistema de Informação de Agravos de Notificação, esteve sujeito a: (i) viés de informação, por registros incorretos ou “ignorados”, mitigado pela exclusão de notificações com dados incompletos; e (ii) viés de seleção, pois somente acidentes oficialmente notificados foram considerados, podendo subestimar eventos em setores informais ou com baixa adesão ao sistema.

### Tamanho do estudo

Não foi realizada estimativa amostral prévia. Utilizou-se a totalidade das notificações de 2015-2019 após critérios de inclusão e exclusão.

### Métodos estatísticos

Os dados foram inicialmente organizados no Microsoft Excel (Microsoft Corporation. Microsoft Excel 365. Redmond, Estados Unidos) e, em seguida, exportados para o software R (R Foundation for Statistical Computing, Viena, Áustria), em que foram realizadas as análises estatísticas, sendo as variáveis categóricas descritas por frequências absolutas e relativas. Para a identificação de padrões nos acidentes de trabalho envolvendo material biológico, foram aplicadas duas técnicas complementares: a análise de correspondência múltipla, utilizada para tratar as variáveis categóricas, e a análise de agrupamento hierárquico, baseada nas componentes principais extraídas da análise de correspondência múltipla.

Devido ao grande volume de dados e às demandas computacionais associadas às técnicas empregadas, foi realizado um sorteio de 3 mil observações para cada ano do período analisado (2015-2019), o que resultou em 15 mil observações para a análise final. Essa estratégia permitiu manter a representatividade da amostra e preservou a robustez dos resultados.

A análise de agrupamento foi implementada utilizando a função HCPC do pacote FactoMineR (9), disponível no R. A determinação do número ideal de agrupamentos foi com base no critério de inércia. Esse critério considerou a redução na inércia total ao formar os agrupamentos, equilibrando homogeneidade intragrupo e simplicidade da solução, o que resultou em divisão otimizada e interpretável dos dados.

Para identificar as variáveis que mais contribuíram para a formação dos agrupamentos, aplicou-se o v-test (9), com nível de significância de 5%, disponível na análise realizada com a função HCPC. O v-test avaliou a associação estatística entre variáveis ou categorias e os agrupamentos formados, medindo o desvio da média observada em relação à média esperada, sob a hipótese nula de que as variáveis ou categorias foram distribuídas aleatoriamente entre os agrupamentos. 

Essa estatística permitiu identificar as variáveis ou categorias mais características de cada grupo, destacando aquelas que estavam significativamente sobrerrepresentadas ou sub-representadas. Valores altos ou baixos (em termos absolutos) do *v-test* indicaram discrepância relevante entre as médias ou proporções observadas e esperadas, sinalizando contribuições significativas para a definição de um agrupamento. Categorias com *v-test* ≤5 foram classificadas como de baixa representatividade; entre 5 e 10, como de representatividade média; e acima de 10, como de alta representatividade. 

Os agrupamentos foram analisados por meio de duas abordagens complementares. A primeira quantificou a proporção de cada categoria em cada agrupamento, enquanto a segunda avaliou a proporção de observações de cada agrupamento em cada categoria.

### Acesso aos dados e métodos de limpeza

Os dados empregados neste estudo são de domínio público e foram disponibilizados pelo Ministério da Saúde. Antes da análise, esses dados passaram por processo de limpeza, com a remoção de duplicatas e a correção de inconsistências, seguido pelas verificações de qualidade para assegurar a integridade e a consistência das variáveis de interesse. O tratamento e a análise dos dados foram conduzidos utilizando o software R. O banco de dados empregado na pesquisa encontra-se disponível em: https://zenodo.org/doi/10.5281/zenodo.15310979 (10).

## Resultados 

Entre 2015 e 2019, o Brasil registrou 69.663 casos de acidentes de trabalho com exposição a material biológico, passando de 12.059 em 2015 para 15.480 em 2019.

A maioria dos acidentados estava na faixa etária 30-39 anos (36,7%), seguida por 16-29 anos (30,7%), com predominância feminina (82,0%) e maior prevalência entre brancos (61,3%). Em termos de escolaridade, 64,9% tinham até o ensino médio, enquanto 27,4% possuíam ensino superior completo (Tabela 1).

**Tabela 1:** Distribuição das características demográficas e ocupacionais (frequências absolutas e relativas) dos casos de acidentes de trabalho com exposição a material biológico. Brasil, 2015-2019 (n=69.663)

Características	n=69.663 (%)
**Ano da notificação**	
2015	12.059 (17,3)
2016	12.739 (18,3)
2017	14.276 (20,5)
2018	15.109 (21,7)
2019	15.480 (22,2)
**Faixa etária** (anos)	
16-29	21.386 (30,7)
30-39	25.539 (36,6)
40-49	14.814 (21,3)
50-59	6.598 (9,5)
60+	1.326 (1,8)
Sexo	
Feminino	57.142 (82,0)
Masculino	12.521 (18,0)
**Raça/cor da pele**	
Branca	42.717 (61,3)
Parda	21.402 (30,7)
Preta	4.872 (7,0)
Amarela	541 (0,8)
Indígena	131 (0,2)
Escolaridade	
Até ensino médio	45.223 (64,9)
Ensino superior incompleto	5.328 (7,6)
Ensino superior	19.112 (27,5)
**Região brasileira**	
Norte	3.152 (4,5)
Nordeste	9.075 (13,0)
Centro-Oeste	6.734 (9,7)
Sudeste	32.013 (46,0)
Sul	18.689 (26,8)
Ocupação	
Auxiliares de laboratório da saúde	1.723 (2,5)
Cirurgiões-dentistas/técnicos de odontologia	4.691 (6,7)
Enfermeiros/técnicos e auxiliares de enfermagem	49.375 (70,9)
Farmacêuticos	825 (1,2)
Médicos	4.636 (6,6)
Trabalhadores dos serviços domésticos/coleta de resíduos, de limpeza e conservação de áreas públicas/tintureiros, lavadeiros	8.413 (12,1)
**Situação de trabalho**	
Formal	61.702 (88,6)
Não formal	7.961 (11,4)
**Tempo de trabalho na ocupação**	
>6 meses	11.880 (17,1)
6-11 meses	7.017 (10,1)
1-2 anos	15.740 (22,6)
3-5 anos	14.164 (20,3)
6+	20.862 (29,9)
**Tipo de exposição**	
Percutânea	61.529 (88,3)
Pele íntegra	21.392 (30,7)
Pele não íntegra	2.758 (4,0)
Mucosa	1.325 (1,9)
**Circunstância do acidentado**	
Administração de medicação	22.273 (32,0)
Punção venosa/arterial	12.176 (17,5)
Descarte e manuseio de materiais	23.436 (33,6)
Procedimento cirúrgico/odontológico/laboratorial	11.778 (16,9)
**Agente causal**	
Agulha/intracath	64.159 (92,1)
Vidro/lâmina/lanceta	5.504 (7,9)
**Uso de equipamento de proteção individual**	
Luva	55.554 (79,7)
Avental	32.489 (46,6)
Óculos	15.271 (21,9)
Máscara	20.129 (28,9)
Proteção facial	3.900 (5,6)
Bota	13.322 (19,1)
Possuía as três doses de vacinação contra hepatite B	66.266 (95,1)
Paciente-fonte conhecido	50.712 (72,8)

O Sudeste concentrou 46,0% dos casos, seguido pelo Sul (26,8%) e pelo Nordeste (13,0%). Profissionais de enfermagem, técnicos e auxiliares representaram 70,9% das notificações. A maioria dos trabalhadores tinha vínculo formal de trabalho (88,6%), e 29,9% estavam na ocupação há seis anos ou mais. A exposição percutânea foi predominante (88,3%), com agulhas e intracaths sendo os principais agentes causais (92,1%) (Tabela 1).

Quanto ao uso de equipamentos de proteção individual, 79,7% usavam luvas, e 46,6%, avental. A cobertura vacinal contra hepatite B foi alta, com 95,1% recebendo as três doses, e 72,8% dos casos envolveram pacientes com fonte conhecida (Tabela 1).

A análise dos agrupamentos evidenciou distinções entre os três grupos identificados, tanto em termos de características demográficas e ocupacionais quanto em relação às circunstâncias dos acidentes com exposição a material biológico (Tabela 2; Figura 1).

**Tabela 2:** Caracterização dos casos de acidentes de trabalho com exposição a material biológico em três grupos (grupo 1 – limpeza e conservação; grupo 2 – enfermagem; grupo 3 – medicina e odontologia), considerando apenas categorias com associação estatisticamente significativa (p-valor<0,05). Brasil, 2015-2019 (n=15.000)

		Grupo 1		Grupo 2		Grupo 3	
Categorias	Geral^a^	Categoria/ grupo^b^	Grupo/ categoria^c^	Categoria/ grupo^b^	Grupo/ categoria^c^	Categoria/ grupo^b^	Grupo/ categoria^c^
**Faixa etária** (anos)							
16-29	30,5	12,8	28,4			20,2	32,7
30-39	36,6	12,6	33,6				
40-49	21,6	16,5	25,8			15,3	17,5
Sexo							
Feminino	82,1	9,8	58,7	74,3	90,5	15,8	69,0
Masculino	17,9	31,6	41,3	35,7	9,5	32,6	31,0
**Raça/cor da pele**							
Branca	62,4	9,8	44,4	68,1	63,0	22,1	73,1
Não branca	37,6	20,3	55,6	66,2	37,0	13,5	26,9
Escolaridade							
Até ensino médio	64,7	20,3	95,4	71,2	68,4	8,5	29,2
Ensino superior incompleto	7,4	4,0	2,1	83,7	9,2	12,3	4,8
Ensino superior	27,9	1,2	2,5	54,2	22,4	44,6	65,9
Região							
Centro-Oeste	10,0	19,8	14,4	59,4	8,8	20,8	11,0
Sudeste	45,3	14,7	48,5			17,6	42,4
Sul	27,2	10,4	20,5	70,0	28,2		
Ocupação							
Auxiliares de laboratório da saúde	2,4	6,0	1,1	80,0	2,9	14,0	1,8
Cirurgiões-dentistas/técnicos de odontologia	6,6	0,9	0,4	8,7	0,9	90,4	31,7
Enfermeiros/técnicos e auxiliares de enfermagem	71,2	2,5	13,2	88,8	93,9	8,6	32,5
Farmacêuticos	1,3	5,6	0,5	82,6	1,6	11,8	0,8
Médicos	6,7	0,2	0,1	6,8	0,7	93,0	33,0
Trabalhadores dos serviços domésticos/coleta de resíduos, de limpeza e conservação de áreas públicas/tintureiros, lavadeiros	11,8	99,0	84,7	0,8	0,1	0,2	0,1
**Tipo de trabalho**							
Formal	88,7	14,6	94,2	71,1	93,6	14,3	67,2
Não formal	11,3	7,0	5,8	38,2	6,4	54,8	32,8
**Tempo de trabalho na ocupação**							
>6 meses	17,4	16,0	20,3				
6-11 meses	10,1	16,7	12,3			14,7	7,9
1-2 anos	22,0	15,9	25,4	65,5	21,4		
6+	30,1	9,5	20,8	69,4	30,9	21,1	33,7
**Tipo de Exposição**							
Percutânea	88,4	14,2	91,2	67,0	87,8		
Mucosa	1,7	4,3	0,5			27,9	2,5
**Circunstância do acidentado**							
Administração de medicação	32,1	0,9	2,1	94,5	45,0	4,6	7,9
Punção venosa/arterial	17,3	0,5	0,6	91,6	23,5	7,9	7,3
Descarte e manuseio de materiais	33,5	39,9	97,1	53,1	26,4	7,0	12,4
Procedimento cirúrgico/odontológico/laboratorial	17,1	0,2	0,2	19,9	5,1	79,9	72,4
**Agente causal**							
Agulha/intracath	92,1	14,1	94,2	69,5	95,0	16,4	80,3
Vidro/lâmina/lanceta	7,9	10,1	5,8	42,9	5,0	47,0	19,7
**Uso de equipamento de proteção individual**							
Luva	79,7	15,4	89,1	61,6	72,8	23,1	97,5
Avental	46,9	10,5	35,8	56,3	39,2	33,2	82,6
Óculos	21,8	8,7	13,7	34,5	11,2	56,8	65,6
Máscara	28,7	9,2	19,2	37,6	16,0	53,2	81,1
Bota	19,2	45,5	63,5	32,4	9,2	22,1	22,5
**Três doses da vacina contra hepatite B**							
Não vacinado	4,9	53,0	19,1	33,6	2,5	13,4	3,5
Vacinado	95,1	11,7	80,9	69,2	97,5	19,1	96,5
**Fonte de exposição conhecida**	72,8	1,6	8,4	76,0	82,1	22,4	86,7

^a^Proporção (%) geral antes do agrupamento; ^b^Proporção (%) da categoria pertencente ao grupo; ^c^Proporção (%) da categoria dentro do grupo.

**Figura 1:** Representatividade das categorias das variáveis analisadas nos grupos de casos de acidentes de trabalho com exposição a material biológico, segundo valores de v-test: ≤5 (baixa representatividade), entre 5 e 10 (representatividade média) e >10 (alta representatividade). Brasil, 2015-2019

Valores de v-test por categoria em cada grupo (A: grupo 1 – limpeza e conservação; B: grupo 2 – enfermagem; e C: grupo 3 – medicina e odontologia); “Inf” indica v-test infinitos decorrentes de denominador zero no cálculo padronizado.

O grupo 1 concentrou majoritariamente trabalhadores do sexo masculino (41,3% diante de 17,9% da proporção geral) com idade entre 40 e 49 anos (25,8%, embora representassem 21,6% da amostra total), não brancos (55,6%) e com escolaridade até o ensino médio (95,4%). Esse grupo foi composto predominantemente por indivíduos vinculados a atividades de limpeza e conservação (84,7%), atuando majoritariamente com vínculo formal de trabalho (94,2%). Os acidentes ocorridos nesse agrupamento foram relacionados ao manuseio de materiais (97,1%) e à exposição percutânea (91,2%). A adesão ao uso de equipamentos de proteção individual, como aventais, máscaras e óculos, foi baixa, assim como a cobertura vacinal contra hepatite B – 19,1% dos integrantes estavam com esquema vacinal incompleto (sendo esse percentual de 4,9% na proporção geral), concentrando 53,0% dos casos totais nessa condição. Houve elevada proporção de trabalhadores que não conheciam a fonte de exposição.

O grupo 2 foi constituído predominantemente por mulheres (90,5%), profissionais de enfermagem (93,9%), com escolaridade até o ensino médio (68,4%) e brancos (63,0%). A maioria desses indivíduos atuava em regime formal (93,5%) e apresentou tempo prolongado na ocupação (30,9% com mais de seis anos de experiência). Esse grupo esteve associado a acidentes durante a administração de medicamentos (45,0%) e revelou alta taxa de cobertura vacinal contra hepatite B (97,5% com três doses), além de elevado grau de conhecimento da fonte de exposição (82,1%). Entretanto, observou-se baixa adesão ao uso de equipamentos de proteção individual, com proporções inferiores em todos os itens avaliados quando comparadas àquelas da amostra geral.

O grupo 3 caracterizou-se pela maior proporção de indivíduos jovens, com idades entre 16 e 29 anos (32,7%), e pela predominância relativa de trabalhadores do sexo masculino em comparação à amostra geral (31,0% versus 17,9%). Destacou-se o elevado percentual de participantes com ensino superior completo (65,9%). Esse grupo foi majoritariamente composto por profissionais das áreas de odontologia e medicina, e cada categoria representou mais de 30,0% dos integrantes do grupo, em contraste com menos de 7,0% de cada categoria na amostra geral. Observou-se expressiva atuação sob regimes de trabalho não formal (32,8%). Predominaram os acidentes relacionados ao uso de materiais perfurocortantes, como vidro e lâminas (19,7%, em comparação a 7,9% na amostra geral), bem como à exposição de mucosas (2,5% diante de 1,7% na amostra total). Ocorreu elevado uso de equipamentos de proteção individual, com frequência superior à média geral. A maioria dos acidentados nesse grupo declarou conhecer a fonte da exposição (86,7%).

## Discussão 

Os achados deste estudo evidenciaram que o risco de acidentes de trabalho envolvendo material biológico no Brasil é condicionado pela interação multifacetada entre fatores sociodemográficos e laborais. Verificou-se a existência de desigualdades marcantes no acesso à proteção e na adoção de práticas preventivas, as quais variam significativamente conforme o grupo ocupacional. A análise permitiu a identificação de três grupos com perfis distintos de trabalhadores acometidos por tais acidentes.

Este estudo apresentou limitações decorrentes do uso de dados secundários, sujeitos a subnotificações e inconsistências nos sistemas de vigilância, o que pode comprometer a precisão dos achados. A ausência de variáveis importantes – como capacitação em biossegurança e tempo de exposição – restringiu a análise dos fatores associados aos acidentes. A heterogeneidade dos contextos laborais e das políticas regionais também pode ter influenciado os resultados, limitando sua generalização. Além disso, a análise se restringiu aos casos notificados no período estudado, sem considerar variações temporais. Apesar dessas limitações, os achados contribuem para a compreensão abrangente dos riscos ocupacionais relacionados à exposição a material biológico e reforçam a necessidade de estratégias preventivas contínuas e efetivas.

O grupo 1 foi composto majoritariamente por homens não brancos, com escolaridade até o ensino médio, atuantes em serviços de limpeza e conservação – perfil semelhante ao observado em um hospital do interior de São Paulo, onde trabalhadores do setor de higiene apresentavam baixa escolaridade, renda reduzida e predominância de autodeclarados pretos e pardos (11). Tal configuração reflete desigualdades estruturais do mercado de trabalho, nas quais pessoas negras, especialmente as menos escolarizadas, ocupam posições mais precárias e expostas a riscos (12). A baixa adesão ao uso de equipamentos de proteção individual, a cobertura vacinal incompleta e o desconhecimento da fonte de exposição observados nesse grupo evidenciam maior vulnerabilidade diante dos riscos ocupacionais com material biológico.

A maioria dos acidentes esteve associada ao manuseio inadequado de materiais perfurocortantes, frequentemente descartados de forma imprópria por profissionais de saúde (13-15). Em seis hospitais de São Luís, foi identificado que 13,57% dos trabalhadores da limpeza sofreram acidentes de trabalho, majoritariamente com materiais perfurocortantes. Indivíduos com ensino médio incompleto apresentaram risco significativamente maior, o que evidenciou o impacto da escolaridade na adesão às práticas de biossegurança (16).

A mitigação desses riscos demanda a disponibilização de dispositivos de segurança apropriados e a oferta contínua de capacitação sobre o uso e descarte correto de materiais perfurocortantes, além da implementação efetiva da segregação adequada dos resíduos de serviços de saúde (17). A não utilização de equipamentos de proteção individual esteve associada ao aumento de cinco vezes na probabilidade de ocorrência de acidentes entre trabalhadores da limpeza urbana em São Luís (16). Em Goiânia, trabalhadores da higienização hospitalar apresentaram altas taxas de uso de luvas e botas de borracha, enquanto a adesão ao uso de avental, máscara e proteção ocular foi substancialmente menor, apesar do reconhecimento de sua importância para a segurança (18). Padrão semelhante foi identificado no grupo 1 desta análise.

O uso adequado dos equipamentos de proteção individual constitui medida fundamental na prevenção de acidentes com material biológico, atuando como barreira física contra contaminações cruzadas e reduzindo substancialmente o risco de infecções ocupacionais (19). A vacinação contra hepatite B representa outra estratégia essencial de proteção para profissionais expostos, sendo eficaz na redução do risco de infecção pelo vírus (20,21). Em Goiânia, aproximadamente um terço dos trabalhadores da higienização hospitalar não havia completado o esquema vacinal, apesar de a importância da imunização ser reconhecida (18) – padrão igualmente observado entre os integrantes do grupo 1 desta investigação.

O grupo 2 foi composto majoritariamente por mulheres brancas, com escolaridade até o ensino médio, atuantes na área de enfermagem, inseridas em regime de trabalho formal e com tempo prolongado de experiência na ocupação. Esse grupo demonstrou elevada cobertura vacinal contra hepatite B e alto grau de conhecimento sobre a fonte de exposição. Apesar desses fatores protetivos, observou-se baixa adesão ao uso de equipamentos de proteção individual, com níveis inferiores aos verificados na amostra geral para todos os itens avaliados. 

Esse grupo apresentou alta frequência de lesões percutâneas com agulhas, as quais ocorreram, majoritariamente, durante procedimentos de administração de medicamentos (22) – uma atividade intrinsecamente associada ao risco de contato com materiais perfurocortantes e fluidos biológicos. Em Minas Gerais, entre outubro de 2014 e maio de 2016, a administração de medicação intramuscular foi identificada como a atividade envolvida em 16,4% das exposições percutâneas notificadas (23). De forma semelhante, em Pernambuco, verificou-se que 62,7% dos acidentes com material biológico estiveram relacionados à administração de fármacos (24).

O grupo 2 concentrou a maior parte dos casos registrados, padrão igualmente observado em nível nacional. Entre 2018 e 2022, 54,4% dos 329.176 acidentes de trabalho com exposição a material biológico ocorreram entre profissionais da enfermagem (25). Este achado foi coerente com a composição majoritariamente feminina da força de trabalho em enfermagem no Brasil. As mulheres representam ampla maioria na categoria, o que contribui para a expressiva participação feminina entre os acidentados (26). Esse perfil é reiterado em estudos regionais: em Minas Gerais, 91,8% dos profissionais expostos eram mulheres da enfermagem (23); em Pernambuco, entre 2009 e 2019, 83,6% dos casos também envolviam profissionais do sexo feminino (24).

O uso de luvas foi relatado entre os profissionais de enfermagem, mas sua frequência foi inferior à observada na amostra geral. A adesão a outros dispositivos de proteção, como óculos e máscaras, mostrou-se ainda mais restrita, o que evidenciou lacunas nas práticas de biossegurança desse grupo (2,3,19). 

Em grande parte dos casos, a fonte de exposição foi identificada, permitindo o adequado seguimento sorológico. Esse achado é corroborado por dados de Minas Gerais, onde o status sorológico do paciente-fonte foi registrado na maioria das notificações de acidentes entre profissionais de enfermagem (23). Além disso, o grupo 2 apresentou cobertura vacinal considerável contra hepatite B. Em Pernambuco, 62,7% dos profissionais acidentados apresentavam o esquema vacinal completo contra esse agravo (24).

O grupo 3 incluiu, de forma proporcionalmente mais expressiva em comparação à amostra geral, profissionais das áreas de odontologia e medicina. Nesse grupo, o manejo inadequado de materiais perfurocortantes – como fragmentos de vidro (possivelmente oriundos de ampolas), lâminas e bisturis – representa vulnerabilidade significativa em termos de biossegurança. Houve alta prevalência de acidentes com materiais perfurocortantes entre trabalhadores da saúde, com maior frequência entre profissionais da odontologia, em razão da realização de procedimentos invasivos e da manipulação constante de instrumentos cortantes (27). Em duas instituições de ensino odontológico no Brasil, 25,3% dos estudantes sofreram acidentes desse tipo durante a formação, com maior incidência nos períodos finais do curso, quando a exposição clínica é mais intensa (28). 

Embora o grupo 3 tenha apresentado proporção mais elevada de homens em comparação à amostra geral, a representatividade feminina permaneceu expressiva. Tal informação não implica associação causal entre gênero e negligência, mas reflete a composição ocupacional específica do grupo analisado. Além disso, esse grupo se destacou pela maior concentração de vínculos empregatícios não formais, incluindo bolsas de estudo, estágios, programas de residência e trabalho autônomo – modalidades contratuais frequentemente observadas entre médicos e cirurgiões-dentistas no contexto brasileiro (29,30).

Apesar da importância do tema, os dados analisados evidenciam lacuna crítica quanto à adesão à profilaxia pós-exposição para o HIV, essencial no manejo imediato de acidentes com risco biológico. A ausência de informações sistematizadas sobre sua indicação, início, continuidade e conclusão dificulta a avaliação das condutas adotadas pelos serviços de saúde, comprometendo o monitoramento institucional e o planejamento de estratégias que garantam acesso equitativo e seguimento adequado. Estudos qualitativos com dados primários são necessários para aprofundar a compreensão dos fatores que influenciam a adesão à profilaxia pós-exposição, incluindo o nível de conhecimento dos profissionais, a estrutura dos serviços e as barreiras institucionais.

Os resultados deste estudo evidenciaram que os acidentes de trabalho envolvendo material biológico no contexto brasileiro resultam de complexa interação entre fatores sociodemográficos, ocupacionais e institucionais. Destacou-se, nesse cenário, a maior vulnerabilidade de grupos específicos, como trabalhadores da limpeza e profissionais de enfermagem, cujas práticas laborais os expõem com maior frequência a situações de risco, especialmente no manuseio de materiais perfurocortantes e na adesão insuficiente às normas de biossegurança. Tal quadro reforçou a necessidade de intervenções estruturadas que articulem ações de educação permanente em saúde, acesso equitativo a equipamentos de proteção individual, fortalecimento das políticas de vigilância em saúde do trabalhador e fiscalização do cumprimento das normas regulamentadoras.
